# Large ore detection in blasting piles using LODM

**DOI:** 10.1038/s41598-025-18474-0

**Published:** 2025-10-06

**Authors:** Xingfan Zhang, Hongdi Jing, Miao Yu, Xin Li, Xiaosong Liu, Zhijian Wang, Yang Cui

**Affiliations:** 1 School of Resources and Security Engineering, University of Science and Technology Beijing, Beijing, 100083 China; 2 Shenyang Institute of Automation, Chinese Academy of Sciences, Shenyang, 110016 China

**Keywords:** Large ore, LODM, Image segmentation, Mask R-CNN, Deep learning, Mineralogy, Petrology

## Abstract

After blasting in an open-pit mine, it has great guiding significance for the subsequent secondary crushing, shovel loading, transportation and other processes to obtain the large ore fragmentations of the blasting pile, which also plays an important role in improving the efficiency and economic benefits of the mine. In this paper, a large ore detection and measurement model LODM based on Mask R-CNN is proposed. After training on our MPBRD1.0 dataset, we compare the detection results with traditional image segmentation algorithms: the K-means clustering algorithm, Canny edge detection algorithm, watershed algorithm and ore image segmentation algorithm based on the U-Net network, which proves that the detection results of the LODM model are more in line with the actual situation. To improve the detection ability of the LODM model, we propose a ResNet34 feature extraction network as the backbone and train ResNet50, ResNet101 and VGG16 at the same time. The results show that the performance of the LODM model can be optimized by using the ResNet34 feature extraction network.

## Introduction

Ore size is a very important aspect in mine blasting, and many scholars have conducted in-depth research on it because it affects the efficiency of subsequent processes, such as loading, transportation, crushing and so on^1–3^. In 1993, Lin and Miller developed an image-based system to obtain the chord length of rock using image processing technology and calculated the cumulative chord length distribution of regularly and irregularly shaped particles using two kernel functions. The last step is to transform the chord length distribution into the size distribution by transforming the equation^4^. In 1988, Lange developed an online real-time system that can collect and process images of rocks on conveyor belts to obtain the chord length distribution and then convert the chord length distribution into the equivalent screening size. Lange provides a method to distinguish bright ore with different particle size distributions^5^.

It is the first step to optimize the blasting parameters to obtain the rock size information of the pile after blasting. At present, the traditional measurement methods of ore size information mainly include the screening method, secondary blasting statistical method, blasting pile direct measurement method, correlation measurement method, empirical method, photogrammetry and so on^6–8^. For example, the screening method will not only consume considerable manpower and material resources but also affect the normal production of the mine, so it is only suitable for laboratory research. The data obtained by the second blasting statistics and the related measurement methods are greatly affected by the equipment, and the accuracy is low. The empirical method relies on experts with experience and is not suitable for large-scale promotion and application.

With the development of image processing technology, many scholars have applied image segmentation technology to the detection of blasting piles. The main image segmentation technologies include the threshold-based segmentation method^9–11^, region-based segmentation method^12–14^, edge-based segmentation method^15,16^ and deep learning-based segmentation method^17–19^. However, the above methods are difficult to use to count the size information of ore after segmentation. For example, Malladi^20^ permuted a watershed transformation approach to generate superpixels efficiently, but this method was not robust enough and time-consuming. Wang^21^ adopted a genetic algorithm in the Otsu threshold image segmentation method; although they reduced the influence of noise and improved the segmentation effect, they still did not solve the problem of oversegmentation and fuzzy boundaries of ore images. Yuan and Duan^22^ proposed an ore particle image segmentation method based on a multilevel strategy. This method could segment ore images with clear boundaries but could not segment images with high luminance points. Above all, we can conclude that they are not suitable for the detection and measurement of the size information of large ores.

In recent years, with the development of deep learning technology, it has appeared in an increasing number of fields, such as face recognition, speech recognition, and automatic driving. Deep learning algorithms were designed to mimic the process of biological learning in the brain^23^, allowing computers to extract features from massive information more effectively. Among many neural network structures, convolutional neural networks (CNNs) have been widely used in image processing due to their efficiency in parameter management and high accuracy.

In the mining field, deep learning technology has not been widely used, which provides novel opportunities the implementation of deep learning in the mining industry^24–26^. The purpose of this study is to use deep learning technology to solve the problem that the labor intensity is high and the ore size cannot be calculated directly in the traditional measurement of fragmentation.

In this study, an LODM model is proposed to detect and measure the size of large ores. After obtaining the pictures of large ores by camera, the pictures are annotated using ‘labelme’, and the dataset is fed into the LODM model for training. The trained model can be used to detect and measure large ores in mines. The main contributions of this study are as follows: (1) The LODM model is established, which is an instance segmentation model specifically used for the detection and measurement of large ores of explosive reactors. (2) It solves the problem that the traditional image segmentation method cannot directly measure the size of the block. (3) In the LODM model, the ResNet34 feature extraction network model is used to optimize the performance of the model. (4) Traditional image segmentation methods are studied, and the segmentation results are compared with those obtained by the LODM model, which proves the superiority of the LODM model. (5) The LODM model with different feature extraction networks is trained, and it is proven that the ResNet34 feature extraction network can make the model performance the best.

## LODM model

### The structure of the LODM model

**Fig. 1:**
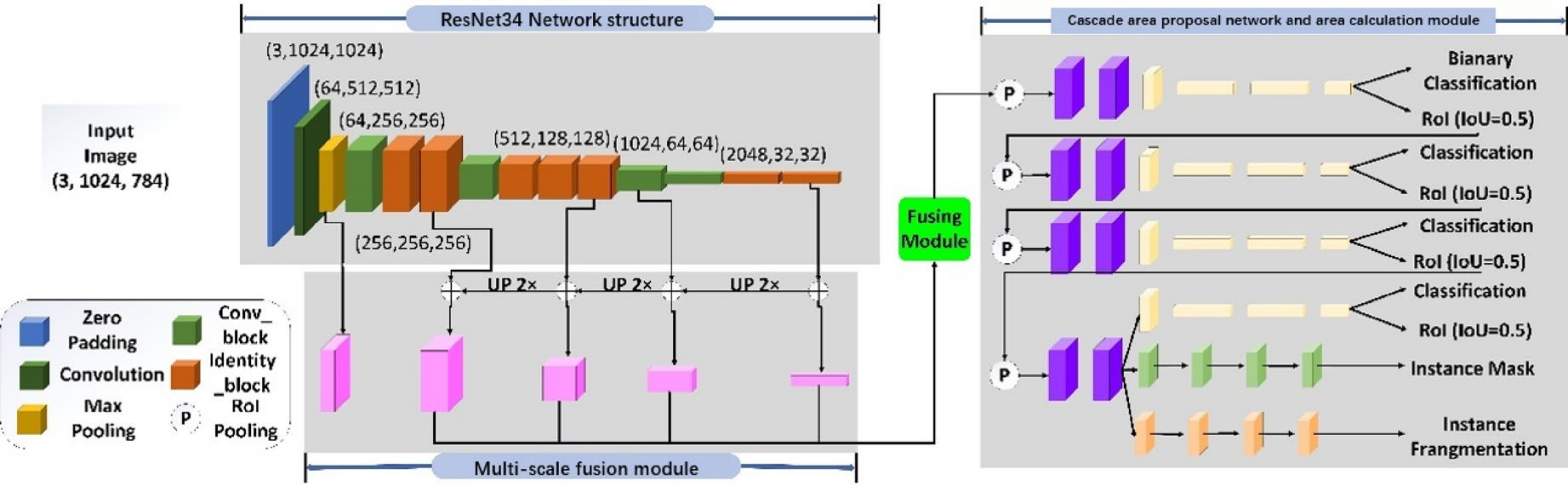
Structure of the LODM model.

**Fig. 2:**
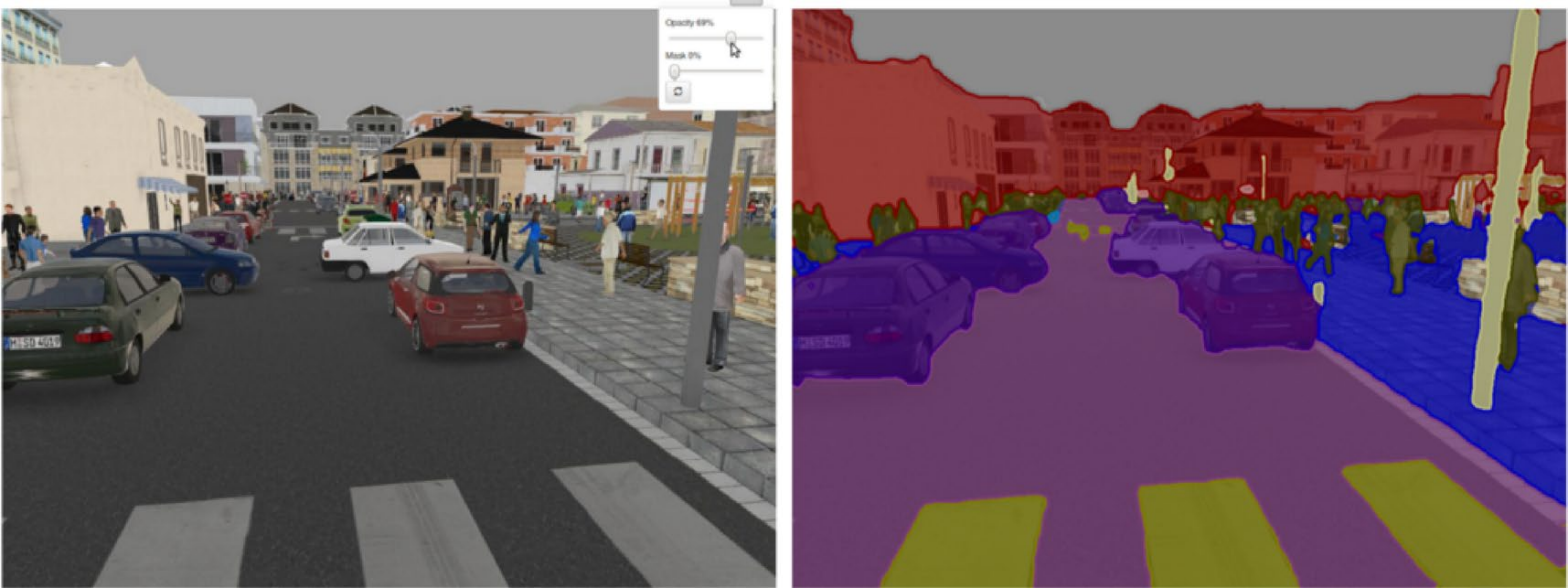
Example of image semantic segmentation mask.

In mine production, blasting is the beginning of mine work. The blasting effect directly affects the subsequent work, such as shovel loading, transportation, crushing and so on. In addition, the blasting effect directly affects the production efficiency and economic benefits of the mine. Therefore, it is economically significant, as its efficiency is directly related to the cost-effectiveness and profitability of mining operations. To optimize the blasting parameters, various evaluation indexes of the blasting effect are needed for reference. There are many indexes for evaluating the blasting effect, such as the large ore rate, front impulse, back impulse, loose coefficient, foundation and so on. Among them, the largest impact on the production efficiency and the most direct index of evaluation effect is the large ore rate. At present, in the field of mining, the measurement method of block rate mainly uses the image analysis method, but the traditional image analysis method cannot give accurate block information. To realize the detection and measurement of the size of large ores, this paper proposes a LODM model based on the Mask-RCNN^27^ instance segmentation model. Figure 1 shows the overall structure of the LODM model. The Mask-RCNN model is a case segmentation model proposed by He K. M in 2017. Because of its high accuracy, the Mask-RCNN model is widely used in the field of case segmentation. However, the Mask-RCNN model can only give the segmentation results of different individuals, as shown in Fig. 2, and cannot output the size information of the target individual. This is because in the field of digital images, the size of the target individual in the image is calculated in pixels, and the actual size of the target individual cannot be calculated. In addition, the feature extraction network used by the Mask-RCNN model is ResNet101, which is composed of 101 network layers. This determines that the ResNet101 network can extract very subtle features in the image, but it also makes the training and detection process very slow. The characteristics of the ResNet101 network make it achieve good results in complex characters, but the ore image has the characteristics of simple structure and single type, so it does not need to use the deep network of ResNet101. Therefore, this paper proposes a feature extraction network ResNet34 with a simple structure. The experimental results show that the accuracy of the ResNet34 network in the field of ore segmentation is almost the same as that of ResNet101, but the speed is greatly improved. To solve the problem that it is difficult to measure the size of ore in the pile blasting image, we propose a method of ore size measurement based on marks. Finally, to make the location of the detection box more accurate, we introduce a cascade area recommendation network based on the coarse-to-fine network structure.

### ResNet34 feature extraction network

The original ResNet architecture was proposed by He et al.^28^. ResNet network can solve the problem of “vanishing gradient” in deep neural networks. This problem is a phenomenon commonly encountered in deep learning, especially when training deep neural networks. It refers to the gradual decrease in gradient as the network hierarchy increases during backpropagation. When the gradient value is very small, the amount of weight updates will also be very small, which can cause the learning process to become very slow.

**Fig. 3:**
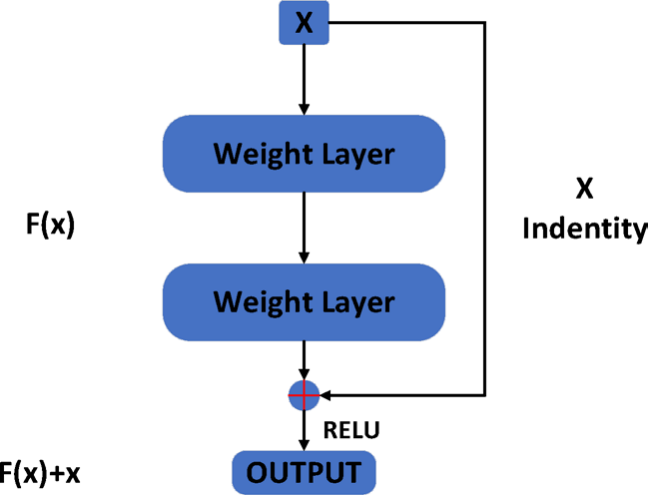
Residual block structure.

The ResNet network is also called the residual network. Resnet is constructed by a residual building block, which includes two kinds of mapping: identity mapping and residual mapping. Figure 3 shows the structure of the residual block. The curve marked with X on the right side of Fig. 3 refers to the identity map, and the f (x) part refers to the residual map. Finally, f (x) + X is output. F (x) + X can be realized by a feedforward neural network with “shortcut connections”. Shortcut connections are the type of connections to skip one or more layers. The “weight layer” in the figure refers to the convolution operation. If the network has reached the optimal level and continues to deepen the network, the residual mapping will become 0, leaving only identity mapping. In this way, the network will be in the optimal state all the time in theory, and the network performance will not decrease with increasing depth. The residual block is composed of several cascaded convolution layers and shortcut connections. After the output values of the two layers are accumulated, the output of the residual block is obtained through the relu activation layer. Multiple residual rocks are connected in series to achieve a deeper network.

The residual structure can be simply written in the following form:

$$x_{l + 1} = x_{l} + F\left( {x_{l} ,W_{l} } \right)$$

Through recursion, the expression of L features of any deep-seated unit can be obtained:

$$x_{L} = x_{l} + \mathop \sum \limits_{i = l}^{L - 1} F\left( {x_{i} ,W_{i} } \right)$$

at is, for any deep element L, the feature $x_{L}$ can be expressed as the feature $x_{l}$ of the shallow element plus a residual function in the form of $\mathop \sum \limits_{i = l}^{L - 1} F$, which indicates that there is residual characteristic between any element L and l.

For backpropagation, the loss function is assumed to be E. According to the chain rule of backpropagation, we can obtain the following results:

$$\frac{\partial \varepsilon }{{\partial x_{l} }} = \frac{\partial \varepsilon }{{\partial x_{L} }}\frac{{\partial x_{L} }}{{\partial x_{l} }} = \frac{\partial \varepsilon }{{\partial x_{L} }}\left( {1 + \frac{\partial }{{\partial x_{l} }}\mathop \sum \limits_{i = l}^{L - 1} F\left( {x_{i} ,w_{i} } \right)} \right).$$

We can find that the derivative can be divided into two parts:

1. Transfer without weight layer $\frac{\partial \varepsilon }{{\partial x_{L} }}$.
2. Transfer through weight layer
  $$\frac{\partial \varepsilon }{{\partial x_{L} }}\left( {\frac{\partial }{{\partial x_{l} }}\mathop \sum \limits_{i = l}^{L - 1} F\left( {x_{i} ,w_{i} } \right)} \right)$$.

The former ensures that the signal can be directly transmitted back to any shallow layer $x_{l}$, and the formula also guarantees that the gradient will not disappear because $\frac{\partial \varepsilon }{{\partial x_{L} }}\left( {\frac{\partial }{{\partial x_{l} }}\mathop \sum \limits_{i = l}^{L - 1} F\left( {x_{i} ,w_{i} } \right)} \right)$ cannot be − 1.

There are two methods of Shortcut Connection:

1. If F(x) and x are added under the same dimension mapping, they are added element by element.
2. If the two dimensions are different, we need to perform a linear mapping for X to match the dimensions.

**Fig. 4:**
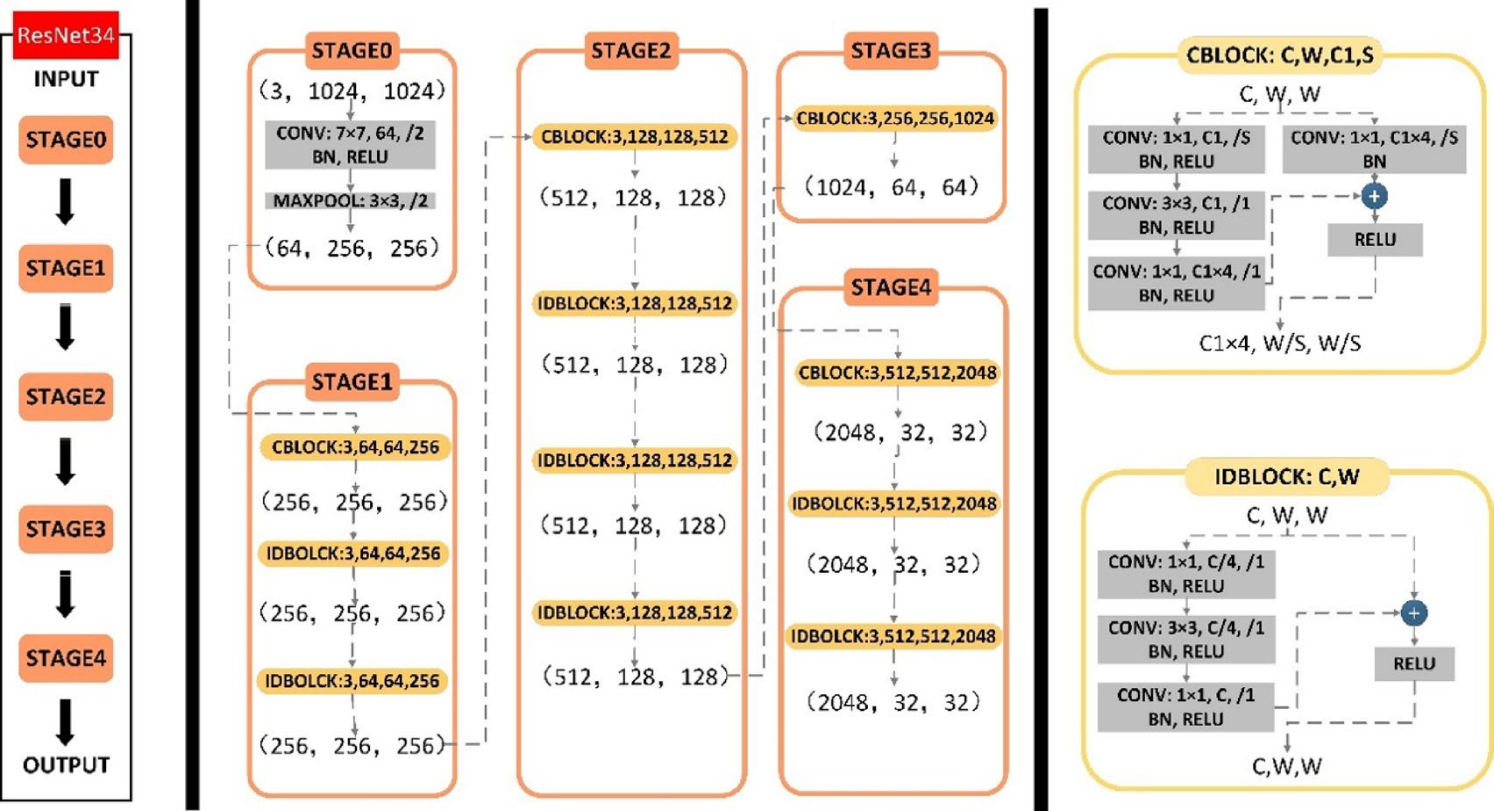
Resnet34 network structure.

It has been pointed out in a previous paper that the popular feature extraction network of the Mask-RCNN model is ResNet101, which is composed of 101 network layers. Therefore, many network layers determine the time-consuming characteristics of its training and detection process. Based on the characteristics of simple structures and single types in ore images, we propose a ResNet34 network structure. The network structure is shown in Fig. 4. In the ResNet34 network structure, we use two kinds of residual rocks. One is that x is convoluted and added to f (x), which is called the conv_ block; the other is that x is directly added to f (x) without the convolution operation, which is called the identity_ block. As shown in Fig. 4.

The two residual rocks adopt the same structure, and both adopt a three-layer convolution structure. After each convolution layer, normalization and activation function processing operations are performed. It should be noted that the activation function processing operation of the third layer is performed after the shortcut connections operation.

There are 34 layers in the ResNet34 network structure. The first layer is the convolution layer and maximum pooling layer, which are used to obtain the first size feature map. Using a conv_block and two identities_block consists of 9 layers obtains the second size feature map. Using a conv_block and three identities_block consists of 12 layers obtains the third size feature map. Using a conv_block consists of 3 layers to obtain the fourth size feature map. Using a conv_block and two identities_ block consists of 9 layers obtains the fifth size feature map.

### Multidimensional feature map fusion mode

**Fig. 5:**
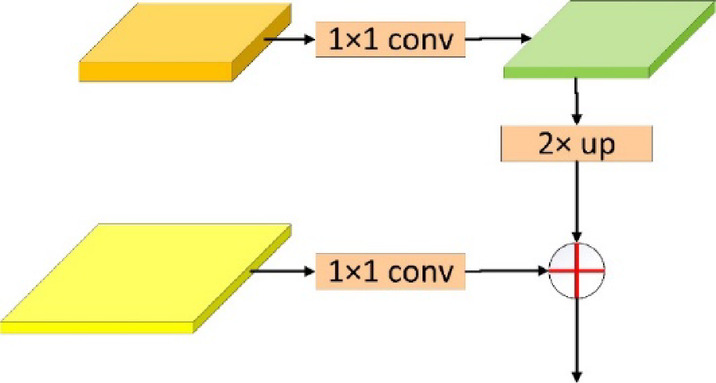
Fusion process of high-dimensional and low-dimensional feature maps in FPN.

In the task of detecting large ore rocks, the difference between the sizes of large ore rocks is a challenging problem. In terms of distance, the size of some ores can reach several meters, while the size of some ores is only approximately tens of centimeters. Generally, the high-dimensional feature map can be used to identify and locate ores with larger block sizes, while the low-dimensional feature map can extract more detailed information to identify and locate ores with smaller block sizes. To improve the ability of model detection and segmentation of different ore rocks, a multiscale feature fusion model named the feature pyramid network (FPN)^29^ is introduced into LODM. In the model, for five different sizes, we extract five different feature maps. Specifically, five feature maps with sizes of 1/4, 1/8, 1/16, 1/32 and 1/64 are extracted for feature fusion. Figure 5 shows the top-down process of two adjacent characteristic graphs in the FPN network. First, the high-dimensional feature graph is divided into a 1 × 1 convolution operation to reduce the number of channels of the high-dimensional feature map. Then, the feature map is subjected to a 2 × upsampling operation to increase the size of the high-dimensional feature map to match the low-dimensional feature map. Finally, the high-dimensional and low-dimensional feature maps are gradually fused through the feature pyramid model. Intuitively, the semantic information of the high-dimensional feature map is compensated for in the low-dimensional feature map with higher resolution to improve the ability of the model to detect targets of different scales.

### Cascaded region proposal network

The region proposal network (RPN)^30^ is a key component of Fast R-CNN, which makes the two-step detection model independent of the traditional target detection algorithm. The training process of RPN is based on a theory called anchor frame, in which the anchor frame can be automatically generated by different aspect ratios to train the RPN network. However, in training, this theory is subject to the imbalance of positive and negative samples. Generally, the RoI can generate approximately 2000 suggested regions. A concept called the intersection union ratio (IOU) is used to distinguish positive and negative samples. The IOU value can determine the ability of the target detector. A lower IOU value means that more samples can pass through the filter in 2000 samples, so more low-quality samples will be generated. By analogy, a high IOU value means that the proportion of high-quality samples in positive samples will be higher, but an overfitting phenomenon may occur, which will reduce the generalization ability of the model. Through our experimental verification, this situation is more serious in the detection of large ores. This is because the noise interference in the ore image will have a great impact on the generation of positive samples, resulting in a significant reduction in the number of positive samples.

**Fig. 6:**
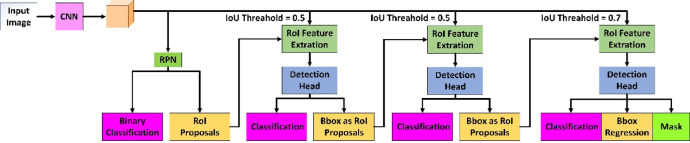
Structure of the cascaded area suggested network.

To improve the detection ability of the LODM model, we add a cascaded RPN structure to the network structure of LODM. Figure 6 shows the overall network structure with cascade detection box regression. Cascaded RPN can improve the detection ability of the model by gradually increasing the value of IOU in the multi-step structure. There is no doubt that the initial suggested region is generated by RPN. In the first step, the IOU value is set to 0.5; in this case, enough regions are judged to be positive samples. In the second and third steps, the IOU values are increased to 0.6 and 0.7, respectively. The disadvantage of this method is that the pooling operation of the ROI will cause dislocation between the ROI and the extracted feature region. Therefore, in this study, we use the RoIAlign method to extract the feature region corresponding to the ROI position.

There are four loss functions in the training process of the LODM model: (1) detection box regression loss function. (2) Target class loss function. (3) The suggested region loss function. (4) Instance partition loss function. In the detection box regression function, it is assumed that the location information of an ROI is b = (b_x_,b_y_,b_w_,b_h_). where b_x_ represents the coordinates in the upper left corner of the detection box and b_w_ and b_h_ represent the width and length of the detection box, respectively. The regression loss function of the detection box is as follows:

$$L_{loc} \left( {a,b} \right) = \mathop \sum \limits_{{i \in \left\{ {x,y,w,h} \right\}}} smooth_{L1} \left( {a_{i} - b_{i} } \right)$$

where $smooth_{L1} \left( \cdot \right)$ represents the loss function of smooth-L1, and the calculation formula is as follows:

$$smooth_{L1} = \left\{ {\begin{array}{*{20}l} {0.5x^{2} ,} \hfill & {\quad \left| x \right| < 1} \hfill \\ {\left| x \right| - 0.5,} \hfill & {\quad otherwise} \hfill \\ \end{array} } \right.$$

The cross entropy loss function (CE) is used for the target species loss function and the suggested region loss function:

$$L_{cls} \left( {h\left( x \right),y} \right) = - \mathop \sum \limits_{i} y_{i} logh_{yi} \left( x \right)$$

where $h\left( x \right)$ is the classification function, which is generally calculated by the softmax function. The formula is as follows:

$$h\left( x \right) = p\left( {y = k|x} \right) = \frac{{e^{{Z_{k} }} }}{{\mathop \sum \nolimits_{j = 1}^{K + 1} e^{{Z_{j} }} }}$$

where z is a k + 1 dimensional vector.

The loss function of instance segmentation calculates the cross entropy loss function of the binary mask of each output object pixel by pixel and finally takes the average value of all values.

The LODM model uses the sum of the above four loss functions as the loss value.

### Block size calculation module

**Fig. 7:**
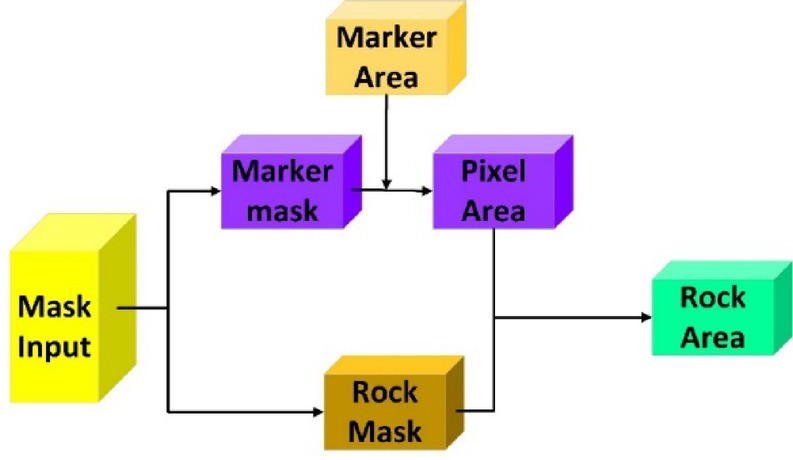
Structure of the LODM prediction module.

At present, the main difficulty of large ore detection is that the true size of large ores cannot be calculated. Based on this premise, this paper proposes a method of large block size measurement based on marks. The size of the electronic image is represented by the resolution, and the resolution of an image represents how many pixels it is composed of. For example, a 1280 × 720 resolution image represents 1280 pixels horizontally and 720 pixels vertically. In other words, the image consists of 1280 × 720 = 921,600 pixels. In practical experience, we know that an image is the mapping of real space on the photo, that is, each image corresponds to a certain area in the real space, and so on, each pixel in each image corresponds to a certain area in the real space. Another key point is that each object in an image is composed of many pixels. Based on the above, we propose a method to calculate the real size of the object in the image, that is, the target size measurement method based on the mark. As shown in Fig. 7.

## Experiment

To verify the proposed LODM bulk detection and size measurement model, we performed many experiments. First, we make a dataset composed of hundreds of images, which is specifically used for the detection and size calculation of large ores. Then, we determine the evaluation index of large block detection. Then, we introduce our experimental details. Finally, we compare the LODM model proposed in this paper with other image segmentation methods and compare the ResNet34 network with other feature extraction networks.

### Dataset preparation

#### Image acquisition and annotation

In this study, we produced a large image dataset named MPBRD1.0. There are hundreds of images in the dataset, and the objects in each image are divided into rocks and markers. Two graduate students annotated the dataset with instance masks by using labelme annotation software. In the process of labelling, we mark the ore rocks that exceed the size of large ores in each picture as Rock1, Rock2, Rock3… and so on until all the large ores in the picture are marked, and the marks in the picture are marked as markers. At the same time, according to the different sizes of large ores, we divide them into three types: super rock (srock), medium rock (mrock) and ordinary rock (nrock). Among them, we define ordinary rock as 2–5 marker sizes, medium rock as 6–8 marker sizes, and super rock as more than 8 marker sizes. As shown in Table 1. In the process of annotation, we count the number of different kinds of large ores in each picture, and some examples are shown in Table 2. Finally, we count the number of different types of rocks in all images and obtain the number distribution statistics of different sizes of rocks in the dataset.

**Table 1:** Criteria for the identification of block types.

Types	Discriminant criteria
marker	One marker
nrock	2–5 markers
mrock	6–8 markers
srock	≥ 9 markers

**Table 2:** Part of the picture annotation information.

Pictures	Types			
	srock	mrock	nrock	Total
90,501	1	11	16	28
90,531	3	9	5	17
90,910	1	9	7	17
90,936	2	2	6	10
91,117	2	8	7	17
91,131	1	10	9	20
91,324	0	3	6	9
91,440	0	4	1	5
91,444	1	4	0	5
91,653	0	11	0	11
Total	11	71	57	139

#### Dataset

**Fig. 8:**
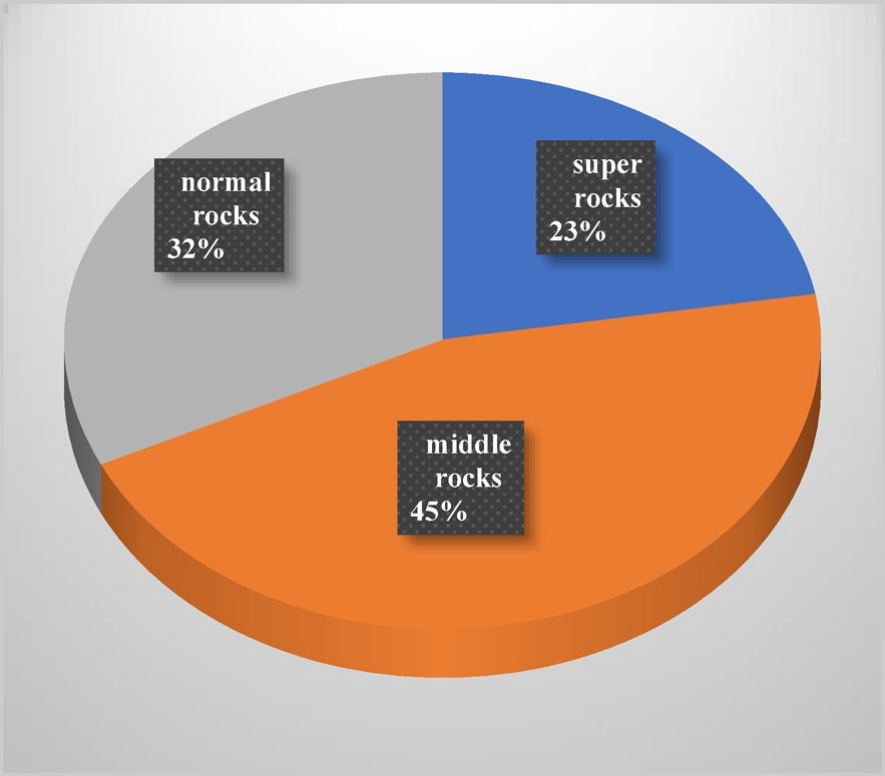
Distribution of three different sizes of rocks.

The structure of the dataset is similar to that of the CoCo dataset. The information folder of each picture is generated by the JSON file of each picture. The folder contains five kinds of information about the picture, namely, the original image, mask code, annotation information and image with mask code. The resolution of the original image is very high, 3840 × 2700. To meet the input requirements of our model, we use the image size conversion script to resize the pictures to 1024 × 768 resolution on the Jupiter compiler. We marked 300 pictures as the training set, and the rest were the test set. Among the labelled rocks, we define 2–5 marker-sized rocks as ordinary rocks, 6–8 marker-sized rocks as medium rocks, and over 8 marker-sized rocks as super large rocks. Table 3 lists the number of rocks of three different sizes in the training set. Figure 8 shows the distribution of the three sizes of rocks in the MPBRD1.0 dataset. In the whole dataset, 32% of the rocks are ordinary rocks, 45% are medium rocks, and the remaining 23% are super rocks.

**Table 3:** Quantity statistics of three different sizes of rocks.

Type	srock	mrock	nrock	Total
Number	328	663	468	1459
Percent	23	45	32	100

The MPBRD10 dataset was compiled and visualised by the authors using an in-house Python script (no public release, hence no name, version or URL can be provided). The script is not publicly available owing to proprietary algorithms that remain under institutional IP restrictions.

#### Data preprocessing

**Fig. 9:**
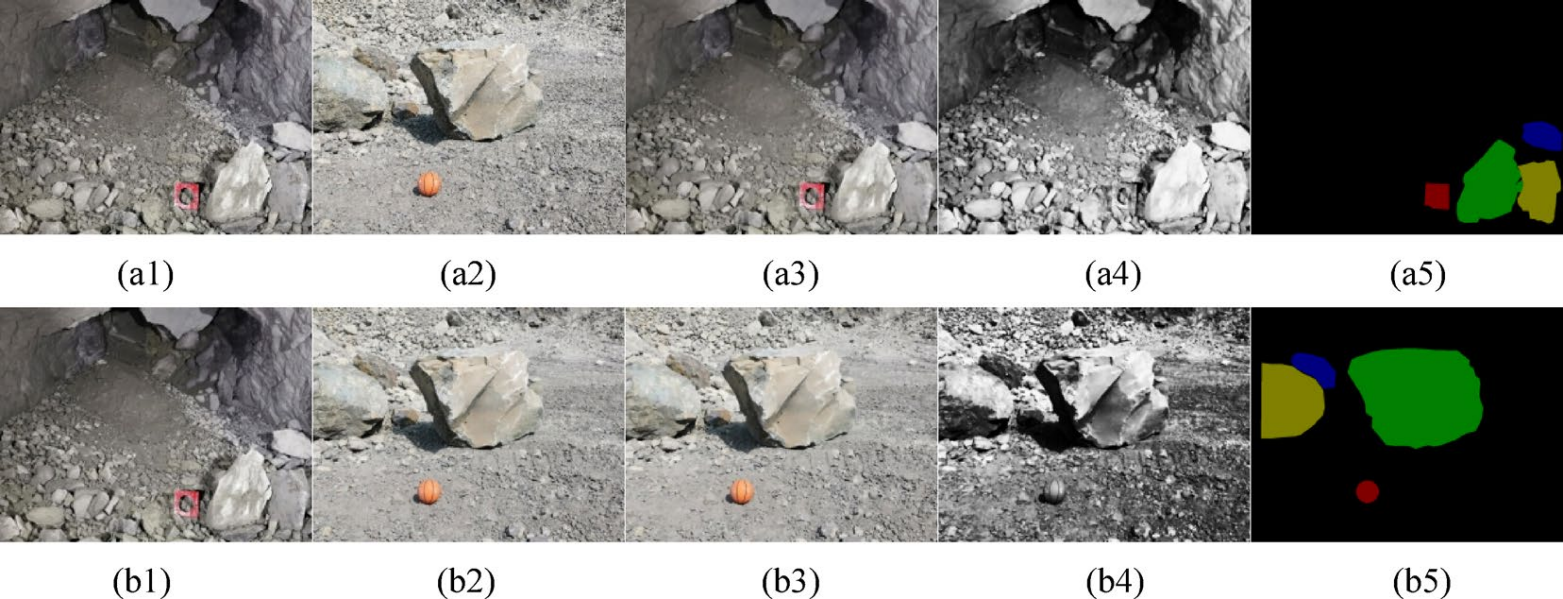
The process of preprocessing.

To solve the influence of noise and improve detection accuracy, we preprocess the image. The steps of preprocessing are as follows: First, we resize the image from 3648 × 2736 to 1024 × 768 because the original image is too large to feed into the network. Second, we process the image with the bilateral filtering method to reduce the influence of noise in the image. Third, we perform histogram equalization on the image; then, the features in the image will be sharper, and the model can learn features more easily. Fourth, we gray the image with the maximum gray scale method to save GPU memory and improve the training speed. The process of reprocessing is shown in Fig. 9.

In Fig. 9a and b represent the open pit mine image and underground mine image, respectively. a1 and b1 are original image of blasting pile, respectively; a2, b2 stand for the size adjusted image; a3, b3 represent the image after bilateral filtering; a4, b4 represent the image after histogram equalization; a5, b5 are mask information of large ores in pile.

### Evaluation method

In our study, we used mean average precision (mAP) and processing time (PT) to evaluate the performance of the model. We use the MS coco method to calculate the map of each rock. The calculation formula is as follows:

$${\text{mAP}} = \frac{{\mathop \sum \nolimits_{class = 1}^{3} \frac{1}{101}\mathop \sum \nolimits_{{r \in \left\{ {0,0.01,0.02, \ldots 1.0} \right\}}} P_{interp} \left( r \right)}}{class}$$

where mAP is the average accuracy, $P_{interp} \left( r \right) = \mathop {\max }\limits_{{\tilde{r}:\tilde{r} \ge r}} p\left( {\tilde{r}} \right)$ represents the highest accuracy rate corresponding to the recall rate r, and *class* represents the type of rock.

mAP is a measure of the accuracy of the target detector, such as Faster R-CNN and SSD. It is the average of the maximum accuracy under different recall rates. The recall rate is the ability of the model to retrieve positive samples correctly. If there are 100 positive samples in the validation set and our model successfully identifies 80 positive samples, then the recall rate of the model is 80%.

The maximum accuracy is the ratio of the real samples among the samples that the model discriminates as positive samples. For example, there are 100 positive samples in our model validation set. However, 80 of the 100 positive samples are real positive samples, and the remaining 20 are actually negative samples. The maximum accuracy of the model is 80%.

The calculation method of the map is to calculate the draw value of the maximum accuracy rate under different recall rates. For example, in the case of 60, 70 and 80% recall rates, the maximum accuracy rates are 70, 80 and 90%, respectively. Then, the map of the model is the average value of the sum of 70, 80, 90%, which is 80%.

### Hyperparameter setting

In the training process, the stochastic gradient descent (SGD) algorithm is used as the optimization algorithm of the model, and the momentum is set to 0.9. The initial learning rate is set to 0.01, the learning rate decay coefficient is set to 1e-4, the epochs of training is set to 108, and the training batch is set to 1. At the beginning of training, we use the decreasing learning rate method to train the model. In the last eight epochs, we use the “multi-step” method to update the learning rate. At the same time, we randomly invert the image vertically to enhance the data. The model is trained in a jupyter notebook using the TensorFlow framework. The training hardware is an NVIDIA Quadro p5000 GPU and a 2.80 GHz Intel (R) core (TM) i9-7960 × GPU.

## Results comparison and discussion

Finally, two contrast methods are proposed to verify the effectiveness and advantage of the LODM model proposed in this study. First, a comparison is made between LODM and traditional image segmentation algorithms. Second, we compare the LODM performance under different feature extraction networks.

### Comparison of the LODM model with traditional image segmentation methods

**Fig. 10:**
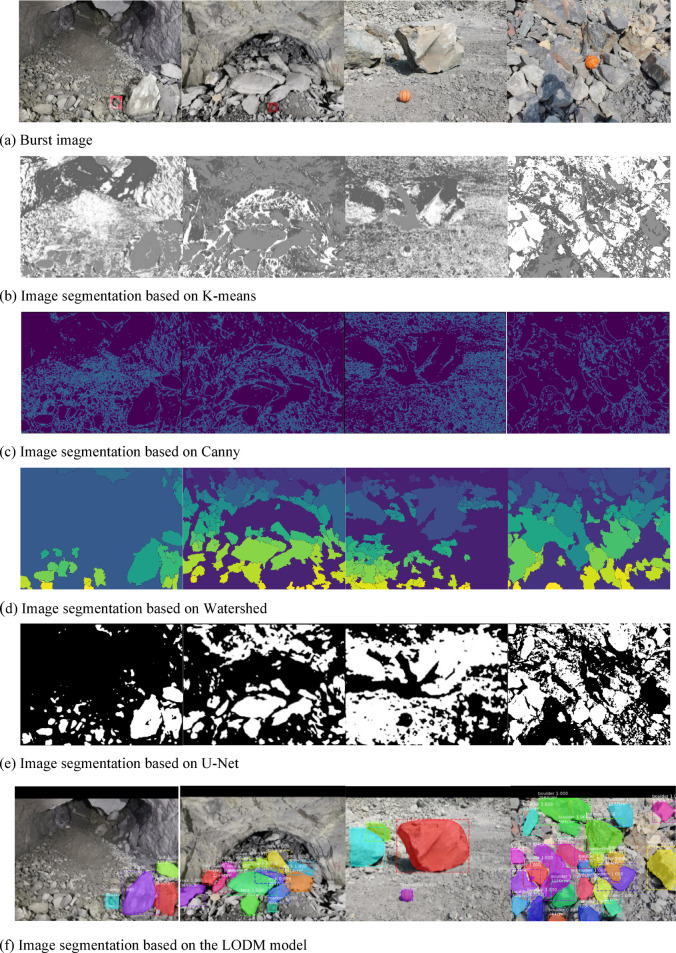
Ore image segmentation effect comparison of five different methods.

To verify the validity of the model, we compare the results of the model with the results of the threshold-based image segmentation method (K-means), edge-based image segmentation (Canny), region-based image segmentation method (Watershed) and U-Net network-based image segmentation, as shown in Fig. 10.

In Fig. 10a, the first two pictures are underground mine blasting piles, and the following two pictures are open pit mine blasting piles. In the above images, the blasting boulder yield is very high. The Fig. 10b represents the segmentation result of the blasting pile image obtained by the K-means method. It can be seen that the K-means method can distinguish different gray areas well. If the gray changes between the ores are not obvious, the segmentation effect of the K-means method is not very good. The Fig. 10c shows the segmentation results of the blasting pile image obtained by the Canny method. It can be seen from the figure that the noise in the blasting pile image has a great influence on the segmentation results, and the boundary of the ore cannot be distinguished. The Fig. 10d illustrates the image segmentation outcomes for pile blasting, achieved through the application of the watershed algorithm. It can be seen that the watershed algorithm cannot segment the real boundary of large ores well. The Fig. 10e shows the image segmentation results of the blasting pile based on U-Net. It can be seen that the U-Net network segmentation method cannot correctly segment open pit mine blasting images, and the ability to distinguish ore and surrounding rocks is not very good.

In addition to their own shortcomings, the four methods of muck pile image segmentation have one common disadvantage: the real size of ore cannot be measured. Therefore, the judgment of large ores can only be judged by human experience without data support. The Fig. 10f shows the detection results obtained by the LODM model.

The detection results shown in Image F are qualitatively superior to those obtained by the other four methods, as evidenced by the higher precision and accuracy metrics used to evaluate the model’s performance. The text displayed on the first line of each detection box in the figure is the category of the target and the score belonging to the category, and the second line is the area of the large ore. Rock represents the type of large ore, a marker represents the type of marker, and the unit of target area is cm^2^. As you can see, the large ores in the figure are accurately identified, and the size of the large ores is also calculated. The segmentation effect of the large rock boundary is better than that of the Canny algorithm, watershed algorithm and U-Net network. Compared with other methods, the segmentation results of large ores in the image have progressed very well, but there is still a gap between the segmentation boundary and the actual ore boundary. The reason for the preliminary analysis is that the samples in the dataset are not sufficient, and more images need to be collected.

### Comparison of ResNet34 and other feature extraction networks

This paper proposes a more compact network, ResNet34, than ResNet101 and ResNet50. There are 34 layers in the ResNet34 network structure. The first layer is the convolution layer and maximum pooling layer, which are used to obtain the first size feature map. A conv_Block and two identities_Block consist of 9 layers to obtain the second size feature map. A conv_Block and three identities_Block consist of 12 layers to obtain the third size feature map. A conv_Block consists of 3 layers to obtain the fourth size feature map. A conv_Block and two identities_Block consist of 9 layers to obtain the fifth size feature map.

**Fig. 11:**
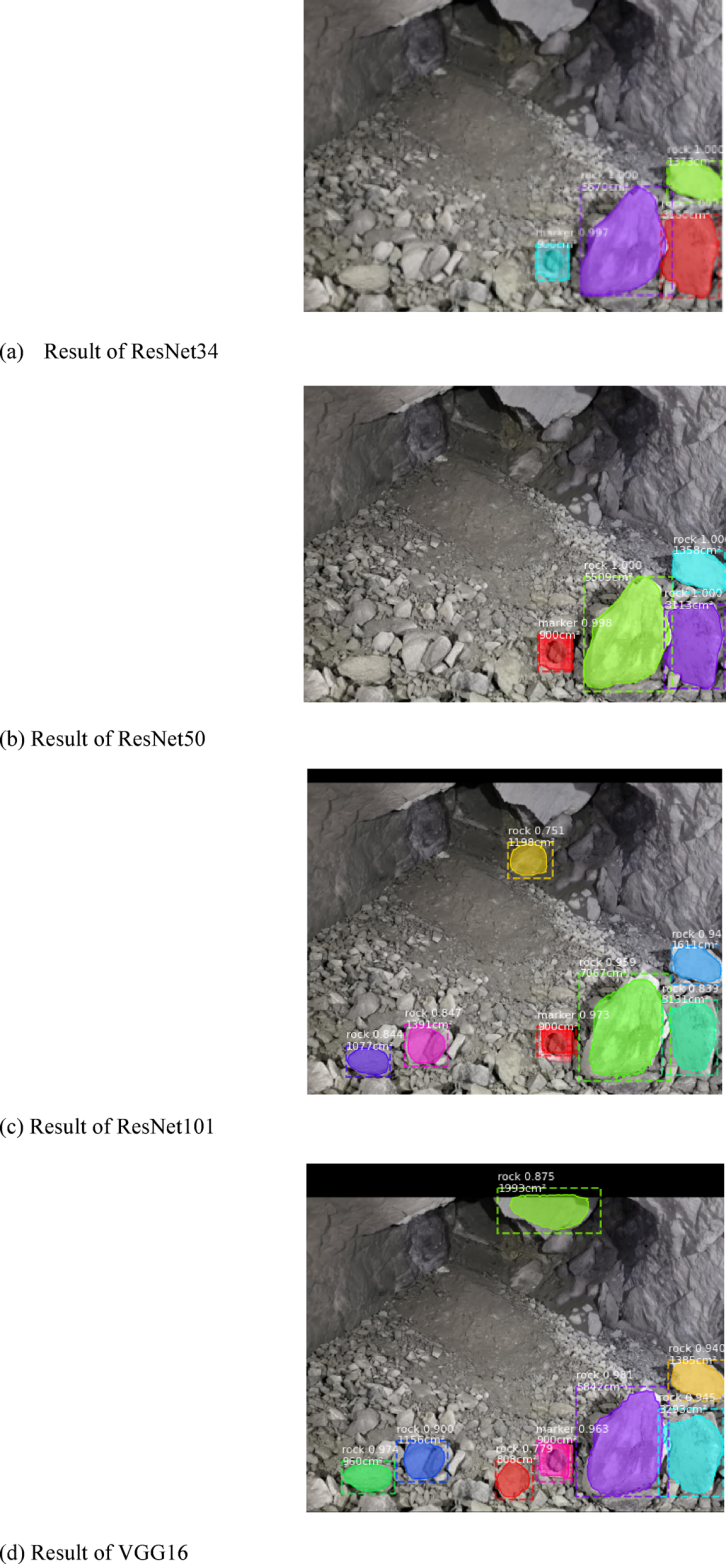
Image processing effect of the blasting pile under different network structures.

To verify the performance of the proposed ResNet34 network in detecting large ores, we trained the ResNet50, ResNet101 and VGG16 networks. The image processing effects of different networks are shown in Fig. 11, and then the image processing time and mAP values of these networks are compared, as shown in Table 4.

**Table 4:** Comparison of four network performance parameters.

Net	Parameter	
	Processing time (s)	mAP
Resnet101	2.61	0.894
Resnet50	2.13	0.837
VGG16	2.01	0.865
ours	1.95	0.895

By comparing the above four graphs, we find that the effect of the image processing of blasting piles obtained by different network structures in the LODM model is similar, which is consistent with the situation that the gap in mAP between the four network structures in the following is not large, which enhances the persuasiveness of this experiment.

The Processing time listed in Table 4 refers to the average forward-pass time (seconds) per 1024 × 768 image on an NVIDIA Quadro P5000 GPU, including Non-Maximum Suppression (NMS) and mask decoding.

It can be seen that in the column of running time, our network reaches the optimal value, and the detection time is the shortest, which is reduced by 25.34% compared with ResNet101, 8.45% compared with ResNet50, and 2.99% compared with VGG16. Compared with ResNet101, the number of layers is greatly reduced, but the accuracy is improved by 0.1%. Compared with ResNet50, the accuracy is improved by 6.48%, and compared with VGG16, the accuracy is improved by 3.35%. Therefore, the LODM model that uses the ResNet34 feature extraction network achieves the optimal performance.

### Rock size calculation results

To validate the accuracy of the block size calculation module (Section “Block size calculation module”), we conducted ground-truth measurements. Thirty test images were randomly selected on site, encompassing a total of 120 pieces of ore, including nrock, mrock, and srock. Physical markers (40cm × 40cm squares) were placed on the blasting pile during image acquisition. The true size of each large ore was manually measured using laser rangefinders, and the pixel-to-cm conversion factor was derived from the marker area in the image.

We evaluated the size estimation error for three ore classes: nrock (2–5 markers), mrock (6–8 markers), and srock (≥ 9 markers). The Mean Absolute Error (MAE) and Mean Absolute Percentage Error (MAPE) were calculated as:

$${\text{MAE}} = \sum \left| {{\text{actual}} - {\text{predicted}}} \right|/{\text{n}}$$

$${\text{MAPE}} = \sum \left| {\left( {{\text{actual}}/{\text{predicted}}} \right) - {1}} \right|/{\text{n}} \times {1}00\%$$

The results are presented in Table 5.

**Table 5:** Size estimation errors by ore class.

Class	Number of samples	MAE (cm)	MAPE (%)	95% CI (cm)
nrock	42	2.1	4.6	[1.6, 2.6]
mrock	48	3.4	3.9	[2.8, 4.0]
srock	30	5.2	3.1	[4.4, 6.0]
Overall	120	3.4	3.9	[2.9, 3.9]

CI, confidence interval of the MAE.

Results demonstrate sub-5% MAPE across classes, validating the marker-based measurement approach. The higher absolute error for srock stems from larger surface irregularities, but lower MAPE confirms robust relative accuracy.

## Conclusions

It is very significant for mine production to quickly identify and measure large ores in blasting piles. To solve the problem of large ore recognition and measurement, this paper proposes a large ore detection model LODM based on case segmentation technology. The establishment of the model mainly includes the following parts: (1) Collecting mine site blasting pile images. (2) Making the dataset by labelme software. (3) Training the LODM model based on the MPBRD1.0 dataset. (4) The training LODM model is used to detect and measure the large ores in the blasting pile.

The experiment shows that compared with the traditional image segmentation method, the detection results of the LODM model proposed in this study not only greatly improve the accuracy of segmentation but also measure the size of the large ores, which can provide theoretical and technical support for actual production. Compared with the traditional feature extraction network, the optimal results are obtained in the running time and average accuracy rate of ResNet34 proposed in this study: compared with ResNet101, the running time of ResNet34 is reduced by 25.34%, and the average mean accuracy rate (mAP) is 0.1%; compared with ResNet50, the operation time is reduced by 8.45%, and the average mean accuracy rate (mAP) is increased by 6.48%; compared with VGG16, the operation time is reduced by 2.99%, and the average mean accuracy (mAP) is increased by 3.35%. In conclusion, the LODM model proposed in this study has achieved good experimental results in research using image segmentation technology to detect large ores, which has strong practical significance and significant application prospects in the field production practice of mines.

## Data Availability

The data that support the findings of this study are available from Ansteel Group but restrictions apply to the availability of these data, which were used under license for the current study, and so are not publicly available. Data are however available from the authors upon reasonable request and with permission of Ansteel Group.

## Code availability

All source code and trained models used in this study are publicly available at https://github.com/jinghongdi/LODM.
